# Hyperspectral imaging as a new diagnostic tool for cervical intraepithelial neoplasia

**DOI:** 10.1007/s00404-023-07171-w

**Published:** 2023-08-14

**Authors:** Lukas Schimunek, Katharina Schöpp, Michael Wagner, Sara Y. Brucker, Jürgen Andress, Martin Weiss

**Affiliations:** 1 Department of Women’s Health, University Hospital Tübingen, 72076 Tübingen, Germany; 2 NMI Naturwissenschaftlich Medizinisches Institut an der Eberhard Karls Universität Tübingen, 72770 Reutlingen, Germany; 3 Section for Plasmamedicine and Medical Technology, Department of Women’s Health, University Hospital Tübingen, Calwerstrasse 7, 72076 Tübingen, Germany

**Keywords:** Hyperspectral imaging, Cervical intraepithelial neoplasia, Contact independent, Marker independent, Cancer prevention, Early detection, Dysplasia

## Abstract

**Purpose:**

Cervical cancer screening by visual inspection with acetic acid (VIA) during colposcopy can be challenging and is highly dependent on the clinical experience of the examiner. Health-care systems lack qualified physicians able to perform the examination in both industrialized and low- and middle-income countries. Previous work has shown the general potential of hyperspectral imaging (HSI) to discriminate CIN from normal tissue, but clinical translation has been limited due to the lack of medically approved HSI systems.

**Methods:**

In this study, we evaluate the feasibility of a commercially available HSI system for CIN detection in a prospective monocentric clinical trial.

**Results:**

By obtaining spectral fingerprints of 41 patients with CIN 1–3 we show that HSI-based differentiation between CIN and normal tissue is possible with high statistical significance. Major spectral differences were seen in the 555–585 wavelength area.

**Conclusion:**

HSI advances tissue differentiation by associating each pixel with high-dimensional spectra and thereby obtains morphological and biochemical information of the observed tissue. Currently available and medically approved HSI systems may represent a contact- and marker-free examiner-independent method for the diagnosis of CIN.

**Supplementary Information:**

The online version contains supplementary material available at 10.1007/s00404-023-07171-w.

## What does this study add to the clinical work

**Table:** 

To overcome current challenges and limitations of cervical cancer screening using visual inspection with acetic acid, this prospective clinical study evaluates hyperspectral imaging (HSI) toward contact- and marker-independent discrimination of cervical neoplasia from normal tissue.	

## Introduction

Cervical intraepithelial neoplasia (CIN) refers to a spectrum of serious precancerous lesions and is classified into three grades based on the extent and severity of cellular and architectural abnormalities. Whereas CIN1 represents mild dysplastic lesions, CIN2 and CIN3 indicate moderate to severe dysplasia. Cervical cancer still is the fourth most common cancer among women worldwide (270,000 deaths/year) and is associated with severe lifelong physical and emotional burdens due to both the disease and radical therapy [1, 2]. Due to this, the identification of CIN lesions within cervical cancer screening programs is crucial. There has been a significant reduction of the cervical cancer incidence in industrialized countries as a result of organized screening programs which include the testing for infections with high-risk human papilloma virus (HPV) types and Pap smear sampling. Suspicious screening results necessitate further colposcopic examination with histological tissue sampling [3, 4], followed by appropriate treatment of detected CIN lesions.

The colposcopic examination and visual inspection with acetic acid (VIA) pose significant challenges and are heavily reliant on the clinical expertise of the examiner. Moreover, access to qualified colposcopy departments remains limited, even in industrialized nations. This issue is further exacerbated in low- and middle-income countries (LMICs), where patients with cervical intraepithelial neoplasia (CIN) lesions rarely have access to skilled colposcopists. Several studies in the literature have reported a wide range of false-negative rates for VIA, ranging from 13 to 69% [5, 6]. Hence, there is an urgent need for contact-free and marker-independent guidance systems to enhance colposcopic accuracy and enable screening by less experienced health-care professionals, such as general practitioners or physician assistants, particularly in LMICs.

In recent years, there have been extensive efforts to apply hyperspectral imaging (HSI) technology in different areas of health care, including tissue differentiation, blood perfusion, wound vascularization and inflammation [7–11]. Within an HSI image, every pixel is assigned to a whole spectrum containing the wavelength-specific reflectance intensity. This results in a three-dimensional (*x*, *y*, *λ*) spectral data cube behind every two-dimensional (*x*, *y*) digital image. There is only a limited number of medically approved HSI systems available, one of which is the TIVITA^®^ Tissue device (Diaspective Vision GmbH, Pepelow, Germany).

The objective of this prospective, monocentric proof-of-principle study was to assess the suitability of the TIVITA^®^ Tissue system for in vivo tissue differentiation of CIN. This investigation extends beyond gynecology and holds relevance for other areas such as the screening of melanoma and premalignant lesions of the oral mucosa.

## Materials and methods

### Study design

The present prospective, monocentric study (DRKS00013486) aimed to investigate the suitability of the TIVITA^®^ Tissue HSI system for differentiation of CIN. The study was performed at the Department for Women’s Health, Tübingen, Germany, in accordance with “The Code of Ethics of the World Medical Association” (Declaration of Helsinki) and was approved by the Ethical Committee of the Medical Faculty of the Eberhardt-Karls-University Tübingen (650-2017BO1). Patients were enrolled in the study after providing informed consent. The key inclusion criteria for study participation were women ≥ 18 years of age with histologically confirmed CIN and an indication for loop electric excision of the transformation zone (LEETZ). CIN was diagnosed by colposcopy-directed biopsy prior to HSI measurement and loop electric excision of the transformation zone (LEETZ) after HSI measurement. To enhance visibility of the CIN lesions, the portio was manually externalized lateral fixation using sutures. HSI was applied after disinfection on a native state portio, followed by VIA, colposcopy and LEETZ under colposcopic guidance. The CIN lesions were histologically re-assessed and mapped to enable the assignment to HSI data. Images were recorded with a distance of 50 ± 5 cm between camera and the tissue surface of enrolled patients positioned in a lithotomy position. For imaging, the operation theater was darkened to prevent accidental distortions by room light. An RGB image was taken after VIA to additionally map the CIN lesions.

### HSI system

For hyperspectral imaging of the portio, the TIVITA^®^ Tissue System (Diaspective Vision GmbH, Pepelow, Germany, CE-0120) was utilized. The camera captures images by detecting the reflected light spectra in the visible and near-infrared wavelength range of 500–995 nm in steps of 5 nm resulting in 100 spectral bands. Homogenic tissue illumination is achieved using six halogen lamps. The screen resolution is 640 × 480 pixel with a spatial resolution of about 0.45 mm/pixel.

### Statistics

For data analysis, HSI images were compared with the high-resolution images of the colposcopic assessment by VIA. Ten respective HSI spectra of 500–995 nm were extracted from the HSI images out of the most representative area of VIA changes and an unsuspicious control area using the TIVITA^®^ software. All spectra were exported to GraphPad Prism version 9.0 (GraphPad Software, San Diego, CA, USA) for further processing. Whole spectra comparisons were conducted using two-way ANOVA of the mean spectral values. Statistical significance was defined by *p* < 0.05. This commonly adopted criterion indicates that any observed differences with a probability of occurring by chance alone of less than 5% are considered statistically significant. Specific *p* values of the different wavelengths between 500 and 995 nm were calculated using Šidák’s multiple comparison test.

## Results

### Patient characteristics

From 06/2018 to 01/2020, we assessed 110 participants for study eligibility. HSI was prospectively applied in 41 patients with histologically proven CIN 1–3 in a controlled clinical trial (DRKS00013486) at the Department of Women’s Health, Tübingen, Germany. CIN was histologically diagnosed by colposcopy-directed biopsy prior to study enrollment. The present prospective, monocentric study included 41 patients aged between 28 and 71 years with histologically confirmed CIN (*n* = 1 patient with CIN 1 (2.4%), *n* = 8 patients with CIN 2 (19.5%), and *n* = 32 with CIN3 (78.1%)). Table 1 shows the patient characteristics of the patients enrolled. The significant difference in the comparison groups was due to the prospective non-randomized nature of the study.

**Table 1:** Patient’s characteristics

No. of patients, *n* (%)	41 (100)
Median age, years (range)	39.6 (28–71)
Histology, *n* (%)	
CIN I	1 (2.4%)
CIN II	8 (19.5%)
CIN III	32 (78.1%)

**Fig. 1:**
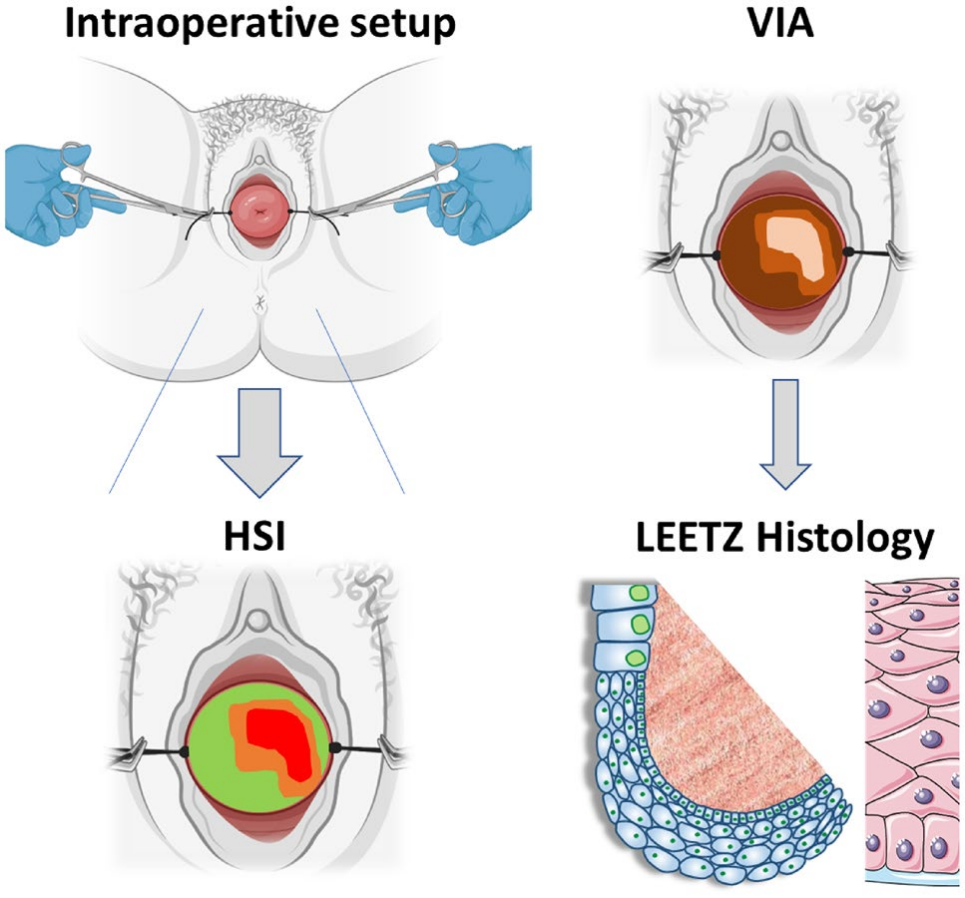
Schematic of the intraoperative setup and experimental workflow. The portio uteri is externalized by lateral sutures. HSI is applied contact and marker independent on native state portio. VIA visualizes the location of the lesion for photodocumentation. CIN lesions are histologically confirmed after LEETZ

The key inclusion criteria for study participation were ≥ 18 years of age, histologically confirmed CIN1-3 with indication for LEETZ, and ectocervical visibility of the lesion margin. Indication for LEETZ was only given in case of either CIN3 lesions, persisting CIN1/2 lesions over 24 months, or by the patient’s compelling wish for therapy (urgent desire to conceive, severe anxiety, and/or significant psychological stress). Prior to HIS measurement, the patients provided written informed consent in accordance with the approved ethical protocol (650-2017BO1) and were informed about the experimental nature of the measurement. CIN1–3 lesions were applied to HSI at native state after disinfection, lateral fixation by sutures, and manual externalization. After contact- and marker-independent HSI measurements, the individual CIN lesions were visualized using colposcopy and VIA, and photodocumented. After performing LEETZ under colposcopic guidance and mapping of CIN localization, the lesion again was histologically confirmed in the conisate. Primary end point was the accuracy of CIN detection and differentiation compared to intraindividual benign regions of the portio according to histological assessment. Figure 1 illustrates the intraoperative setup and experimental workflow of this study.

### Statistical comparison of absorption per wavelength between CIN lesions and healthy tissue

**Fig. 2:**
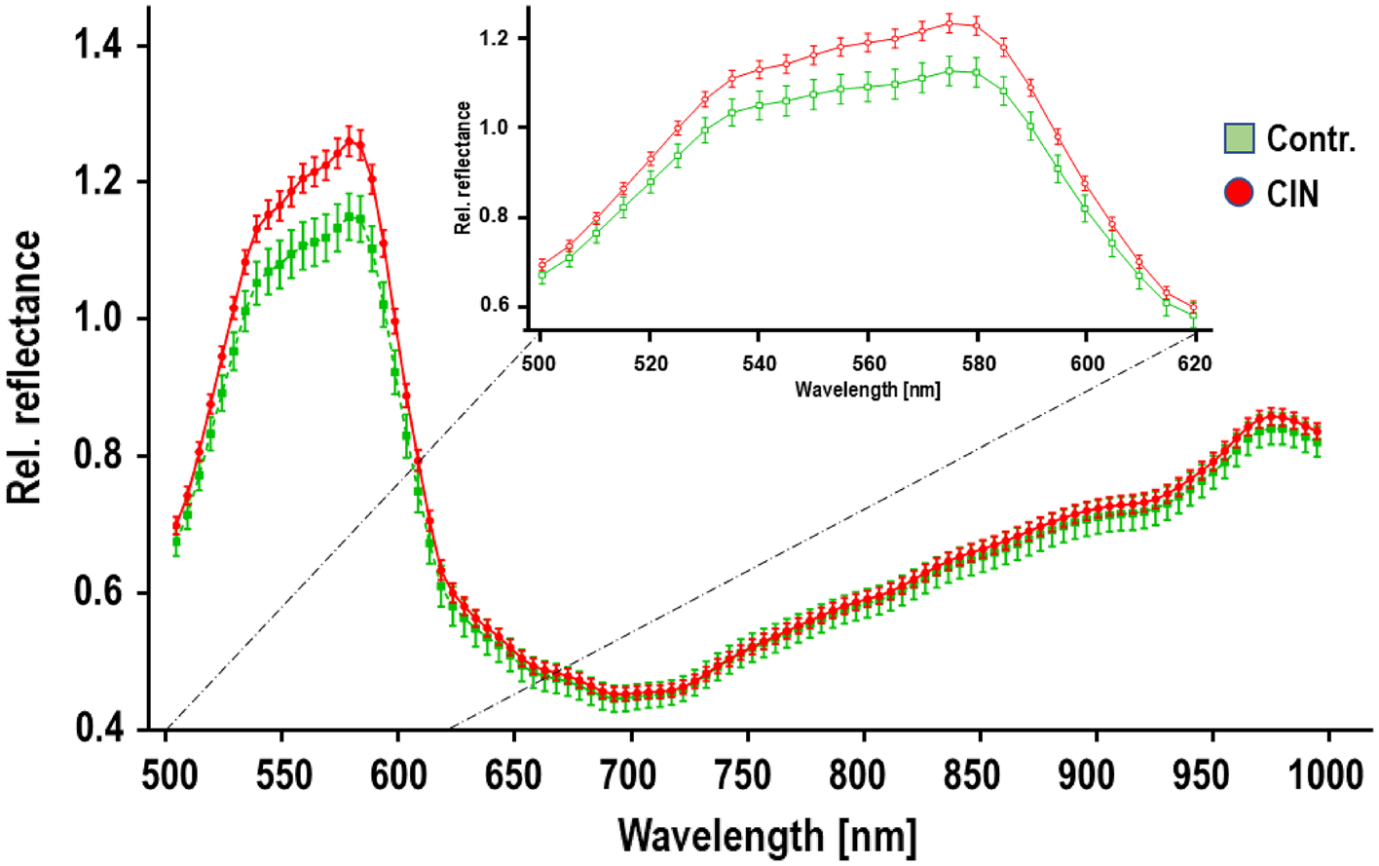
Obtained HSI spectra of CIN lesions vs. control. Graphs depict the mean accumulative reflectance of CIN lesions (red) as well as healthy control areas (green) ± 1 standard deviation (SD) with wavelengths from 500 to 995 nm on the x-axis and relative reflectance in arbitrary units on the y-axis

The comprehensive analysis of the entire wavelength spectrum, ranging from 500 to 990 nm, revealed substantial and statistically significant differences between CIN lesions and healthy tissue of the portio (*p* value < 0.0001, obtained from a two-way ANOVA analysis; Supplementary Table 1). This broad examination of hyperspectral information allowed for a more comprehensive evaluation of the spectral characteristics and highlighted the potential of utilizing a wide range of wavelengths for the discrimination of abnormal tissue (Fig. 2).

Deeper analysis of the obtained HSI spectra highlights the specific wavelength range of 555–585 nm as particularly sensitive for distinguishing between CIN lesions and healthy cervical tissue. The absorption of this wavelength region demonstrated statistically significant differences represented by calculated *p* values < 0.05 by using a Student’s *T* test, indicating that the spectral characteristics within this range can serve as reliable indicators of abnormal tissue (Table 2). Furthermore, the most pronounced discrepancy was observed at 575 nm, with a remarkably low *p* value of 0.0048. This specific wavelength exhibited the highest discriminatory power, suggesting that it could potentially be utilized as a targeted marker for the detection and diagnosis of CIN lesions during colposcopy procedures.

**Table 2:** Wavelengths with statistically significant hyperspectral differences between lesions and benign tissue regions

Wavelength (nm)	Relative absorption CIN lesions	Relative absorption controls	Predicted (LS) mean difference	Standard error of difference	Significance	*p* value
555	1.205	1.106	0.09906	0.02729	*	0.0280
560	1.215	1.112	0.1036	0.02729	*	0.0147
565	1.225	1.118	0.1065	0.02729	**	0.0095
570	1.242	1.133	0.1096	0.02729	**	0.0059
575	1.260	1.149	0.1110	0.02729	**	0.0048
580	1.254	1.146	0.1088	0.02729	**	0.0067
585	1.204	1.102	0.1021	0.02729	*	0.0182

## Discussion

Colposcopy, as the current gold standard, plays a vital role in evaluating cervical abnormalities. It has significantly contributed to cervical cancer screening, diagnosis, and decision-making processes. By providing enhanced visualization of the cervix, colposcopy, combined with VIA and iodine staining, enables the identification of subtle changes and abnormalities. It facilitates targeted biopsy of suspicious areas and provides visual guidance during treatment procedures, leading to improved accuracy of diagnosis and treatment. Through early detection and evaluation of CIN, colposcopy plays a critical role in preventing the progression to invasive cervical cancer. However, it is important to acknowledge that the accuracy and interpretation of colposcopic findings can vary among practitioners. Generally, the highest accuracy is achieved by highly experienced colposcopists, which presents a challenge for ensuring universal access to quality healthcare services. While colposcopy demonstrates good sensitivity, it exhibits only moderate specificity. This often results in frequent and burdensome biopsy procedures for patients [12]. Consequently, there is a need for contact- and marker-free examiner-independent technologies to remove these limitations. The tissue discrimination and decision-making aid by HSI could decisively improve patient care in both, industrialized as well as LMICs and would be relevant for other medical specialties beyond gynecology.

In recent years, several groups have investigated spectroscopic technologies just like fluorescence spectra and reflectance spectra [13, 14]. Besides spectroscopy of native tissue, there are emerging systems that specifically target the changes in spectral signatures following the application of acetic acid. These systems aim to detect the temporarily modified scattering properties of abnormal epithelium, enabling improved detection and characterization of cervical abnormalities. Some of these technologies have already been implemented in clinical settings and are currently being investigated in clinical trials [15]. Also, the combination of sensors. e.g.. reflectance and fluorescence spectroscopy was evaluated in previous studies [16]. Even reflectance recognition by smartphone was recently evaluated in patients [17]. All of these technologies are based on the application of different marker substances onto the cervix uteri before CIN measurement. Nevertheless, the development of a marker-independent system that relies solely on the spectral characteristics of cervical tissue would offer several advantages. Such a system would simplify the examination procedure by eliminating the need for acetic acid application and its associated influencing factors, which can lead to enhanced or unspecific tissue reactions. Additionally, a marker-independent system could be adapted for use in non-cervical tissues, including the vulva, where the predictive value of acetic acid application is less reliable [17].

Several research groups have explored the feasibility of employing a hyperspectral approach to differentiate between normal and dysplastic cervical tissue, by investigating the spectral signatures of cervical tissue across a range of wavelengths [18–21]. Already in 2001, Ferris et al. described reflectance measurements after an HSI probe was placed on the ectocervix [18]. Comparison of HSI and PAP smear sensitivity and specificity showed equal specificity (70%) for both tests, whereas the sensitivity of HSI was 97%, compared to 72% for the Pap smear. In 2007, a study by Mourant et al. showed the spectroscopic results of 29 patients, compared with histopathology results and using ROC curves, MANOVA, and logistic regression. They found three spectroscopic parameters being statistically different for HSIL compared with low-grade lesions and normal tissue enabling CIN discrimination with 100% sensitivity and 80% specificity. In 2015, the research group led by Zheng et al. obtained hyperspectral data cubes within the wavelength range of 600–800 nm [21]. They further processed the data using a wide gap second derivative analysis method. By this, Zheng et al. found that specific wavelengths, namely 620 nm, 696 nm, and 772 nm, were sufficient for tissue classification, achieving optimal separability between different tissue types. The authors successfully demonstrated the feasibility of classifying cervix tissue into three categories: normal, inflammation, and high-grade lesion. It should be noted, however, that this proof-of-concept study was conducted on a limited scale, involving only three patients.

Despite recent successes in tissue differentiation using HSI, the clinical application and further research in this field face limitations. Current challenges include the bulky setup and high costs of experimental HSI systems, as well as the time-consuming nature of data acquisition and processing. The availability of medically approved HSI systems that could be easily applied for evaluating the technology remains limited. These factors impede the broader adoption and exploration of HSI in medical settings. To overcome these limitations, we acquired hyperspectral cubes using the TIVITA^®^ Tissue System, which is a medically approved device that measures in the wavelength range of 500–995 nm. Initially designed for wound and perfusion diagnostics, the TIVITA^®^ Tissue System has demonstrated great potential in the field of tissue differentiation, as indicated by a recent study [22]. In this study, Studier-Fischer et al. collected an HSI dataset from the visceral organs of 46 pigs and obtained 9059 HSI images in total. Based on this, the authors developed a comprehensive tissue atlas with spectral fingerprints of 20 distinct porcine organs and tissue types. Utilizing mixed model analysis and deep neural networks, the authors further demonstrated the feasibility of fully automated tissue differentiation across the 20 organ classes with an accuracy exceeding 95%.

The primary objective of the current investigation was to assess the feasibility of utilizing the TIVITA^®^ Tissue System for the differentiation of CIN lesions. However, a significant constraint associated with this system is the limited tissue–camera distance of 50 cm, which hinders its applicability during routine colposcopy procedures. Due to this, HSI measurement was performed intraoperatively after lateral fixation and manual externalization of the cervix by sutures. Even in this setup the actual area of the portio remained very small not exceeding 5–10% of the displayed HSI area. Despite these limitations, the analysis of the hyperspectral information revealed statistically significant differences between CIN lesions and healthy tissue especially within the wavelengths of 555–585 nm. This finding suggests that the TIVITA^®^ Tissue System and furthermore the HSI technology in general is principally feasible to discriminate between dysplastic and healthy cervical tissue and should be evaluated in further studies. An endoscopic version of the TIVITA^®^ Tissue System is currently under development, aiming to improve the resolution through reduced tissue-camera distance. This innovative advancement holds the potential to significantly improve the system's applicability during colposcopy procedures. Overcoming current technical limitations by enabling closer proximity to the tissue of interest, the endoscopic version would enhance not only the diagnostic capabilities but also the interpretability of measurement results. Focusing the resolution on the target tissue will enable research on the detection and discrimination of other human diseases such as chronic inflammatory diseases as well as tissue and organ recognition to enhance intraoperative safety.

Besides the promising results of this study, it involves several limitations based on the technical issues described above and the performed data analysis. In the clinical setup it is not practical to externalize the cervix uteri by lateral sutures to enable accessibility for HSI. HSI sensors must be able to enter the vaginal cavity to achieve high-resolution HSI images. The extraction of HSI spectra by hand based on the information of colposcopy and VIA lacks reproducibility and may be a source of bias. The statistical comparisons on mean values using two-way ANOVA and Šidák’s multiple comparison test may entail overperformance of the results. In future, the clustering of hyperpixel information by machine learning algorithms could be an interesting strategy to illustrate HSI-based differences in tissue composition. Further limitations of this proof-of-principle study include the small sample size of 41 patients and the uneven distribution of included CIN lesions. Large-scale confirmatory clinical trials involving diverse patient populations, along with the integration of advanced image analysis techniques as well as machine learning algorithms and neuronal networks, will be crucial to optimize the clinical utility of HSI in the detection and management of cervical abnormalities. Further confirmatory prospective clinical studies are under development to verify the results of this study with second generation HSI devises overcoming the above described technical limitations.

### Supplementary Information

Below is the link to the electronic supplementary material.

Supplementary file1 (DOCX 28 KB)

## Data Availability

Original data is shown in this manuscrip.
