# Supplementary material for: Hyperspectral imaging as a new diagnostic tool for cervical intraepithelial neoplasia
Source: Arch Gynecol Obstet. 2023 Aug 14;308(5):1525–30. doi: 10.1007/s00404-023-07171-w (PMC10520109; doi:10.1007/s00404-023-07171-w)
Supplement: Supplementary file 1 — Supplementary file1 (DOCX 28 KB) [file 404_2023_7171_MOESM1_ESM.docx]

| Wavelength (nm) | Relative Absorption CIN-Lesions | Relative Absorption Controls | Predicted (LS) mean difference | Standard Error of difference | Significance | p-Value |
| --- | --- | --- | --- | --- | --- | --- |
| 500 | 0,6959 | 0,6719 | 0,02402 | 0,02729 | ns | >0,9999 |
| 505 | 0,7399 | 0,7123 | 0,02768 | 0,02729 | ns | >0,9999 |
| 510 | 0,8042 | 0,7700 | 0,03423 | 0,02729 | ns | >0,9999 |
| 515 | 0,8735 | 0,8301 | 0,04344 | 0,02729 | ns | >0,9999 |
| 520 | 0,9437 | 0,8897 | 0,05400 | 0,02729 | ns | 0,9926 |
| 525 | 1,015 | 0,9512 | 0,06393 | 0,02729 | ns | 0,8553 |
| 530 | 1,083 | 1,010 | 0,07217 | 0,02729 | ns | 0,5603 |
| 535 | 1,131 | 1,052 | 0,07951 | 0,02729 | ns | 0,3011 |
| 540 | 1,152 | 1,068 | 0,08372 | 0,02729 | ns | 0,1942 |
| 545 | 1,165 | 1,079 | 0,08623 | 0,02729 | ns | 0,1462 |
| 550 | 1,186 | 1,094 | 0,09194 | 0,02729 | ns | 0,0727 |
| 555 | **1,205** | **1,106** | **0,09906** | **0,02729** | ***** | **0,0280** |
| 560 | **1,215** | **1,112** | **0,1036** | **0,02729** | ***** | **0,0147** |
| 565 | **1,225** | **1,118** | **0,1065** | **0,02729** | ****** | **0,0095** |
| 570 | **1,242** | **1,133** | **0,1096** | **0,02729** | ****** | **0,0059** |
| 575 | **1,260** | **1,149** | **0,1110** | **0,02729** | ****** | **0,0048** |
| 580 | **1,254** | **1,146** | **0,1088** | **0,02729** | ****** | **0,0067** |
| 585 | **1,204** | **1,102** | **0,1021** | **0,02729** | ***** | **0,0182** |
| 590 | 1,110 | 1,020 | 0,09010 | 0,02729 | ns | 0,0917 |
| 595 | 0,9953 | 0,9204 | 0,07494 | 0,02729 | ns | 0,4540 |
| 600 | 0,8862 | 0,8271 | 0,05912 | 0,02729 | ns | 0,9538 |
| 605 | 0,7913 | 0,7463 | 0,04500 | 0,02729 | ns | >0,9999 |
| 610 | 0,7031 | 0,6703 | 0,03280 | 0,02729 | ns | >0,9999 |
| 615 | 0,6304 | 0,6068 | 0,02362 | 0,02729 | ns | >0,9999 |
| 620 | 0,5970 | 0,5774 | 0,01956 | 0,02729 | ns | >0,9999 |
| 625 | 0,5774 | 0,5601 | 0,01726 | 0,02729 | ns | >0,9999 |
| 630 | 0,5597 | 0,5445 | 0,01512 | 0,02729 | ns | >0,9999 |
| 635 | 0,5452 | 0,5317 | 0,01352 | 0,02729 | ns | >0,9999 |
| 640 | 0,5325 | 0,5201 | 0,01235 | 0,02729 | ns | >0,9999 |
| 645 | 0,5170 | 0,5058 | 0,01118 | 0,02729 | ns | >0,9999 |
| 650 | 0,5007 | 0,4906 | 0,01015 | 0,02729 | ns | >0,9999 |
| 655 | 0,4894 | 0,4800 | 0,009361 | 0,02729 | ns | >0,9999 |
| 660 | 0,4837 | 0,4748 | 0,008811 | 0,02729 | ns | >0,9999 |
| 665 | 0,4795 | 0,4710 | 0,008494 | 0,02729 | ns | >0,9999 |
| 670 | 0,4749 | 0,4666 | 0,008307 | 0,02729 | ns | >0,9999 |
| 675 | 0,4685 | 0,4604 | 0,008116 | 0,02729 | ns | >0,9999 |
| 680 | 0,4602 | 0,4524 | 0,007756 | 0,02729 | ns | >0,9999 |
| 685 | 0,4524 | 0,4452 | 0,007154 | 0,02729 | ns | >0,9999 |
| 690 | 0,4480 | 0,4415 | 0,006505 | 0,02729 | ns | >0,9999 |
| 695 | 0,4480 | 0,4421 | 0,005935 | 0,02729 | ns | >0,9999 |
| 700 | 0,4498 | 0,4444 | 0,005377 | 0,02729 | ns | >0,9999 |
| 705 | 0,4511 | 0,4464 | 0,004735 | 0,02729 | ns | >0,9999 |
| 710 | 0,4517 | 0,4477 | 0,004028 | 0,02729 | ns | >0,9999 |
| 715 | 0,4538 | 0,4505 | 0,003376 | 0,02729 | ns | >0,9999 |
| 720 | 0,4587 | 0,4560 | 0,002652 | 0,02729 | ns | >0,9999 |
| 725 | 0,4669 | 0,4651 | 0,001852 | 0,02729 | ns | >0,9999 |
| 730 | 0,4779 | 0,4768 | 0,001160 | 0,02729 | ns | >0,9999 |
| 735 | 0,4894 | 0,4886 | 0,0007961 | 0,02729 | ns | >0,9999 |
| 740 | 0,4997 | 0,4990 | 0,0007762 | 0,02729 | ns | >0,9999 |
| 745 | 0,5092 | 0,5082 | 0,001008 | 0,02729 | ns | >0,9999 |
| 750 | 0,5179 | 0,5163 | 0,001539 | 0,02729 | ns | >0,9999 |
| 755 | 0,5256 | 0,5233 | 0,002268 | 0,02729 | ns | >0,9999 |
| 760 | 0,5331 | 0,5302 | 0,002869 | 0,02729 | ns | >0,9999 |
| 765 | 0,5410 | 0,5377 | 0,003299 | 0,02729 | ns | >0,9999 |
| 770 | 0,5487 | 0,5451 | 0,003658 | 0,02729 | ns | >0,9999 |
| 775 | 0,5563 | 0,5523 | 0,004002 | 0,02729 | ns | >0,9999 |
| 780 | 0,5638 | 0,5594 | 0,004353 | 0,02729 | ns | >0,9999 |
| 785 | 0,5711 | 0,5663 | 0,004796 | 0,02729 | ns | >0,9999 |
| 790 | 0,5777 | 0,5724 | 0,005319 | 0,02729 | ns | >0,9999 |
| 795 | 0,5833 | 0,5775 | 0,005813 | 0,02729 | ns | >0,9999 |
| 800 | 0,5879 | 0,5818 | 0,006119 | 0,02729 | ns | >0,9999 |
| 805 | 0,5926 | 0,5864 | 0,006239 | 0,02729 | ns | >0,9999 |
| 810 | 0,5989 | 0,5925 | 0,006463 | 0,02729 | ns | >0,9999 |
| 815 | 0,6074 | 0,6004 | 0,006913 | 0,02729 | ns | >0,9999 |
| 820 | 0,6169 | 0,6095 | 0,007448 | 0,02729 | ns | >0,9999 |
| 825 | 0,6264 | 0,6185 | 0,007931 | 0,02729 | ns | >0,9999 |
| 830 | 0,6354 | 0,6269 | 0,008492 | 0,02729 | ns | >0,9999 |
| 835 | 0,6435 | 0,6343 | 0,009168 | 0,02729 | ns | >0,9999 |
| 840 | 0,6503 | 0,6405 | 0,009806 | 0,02729 | ns | >0,9999 |
| 845 | 0,6561 | 0,6457 | 0,01039 | 0,02729 | ns | >0,9999 |
| 850 | 0,6615 | 0,6506 | 0,01086 | 0,02729 | ns | >0,9999 |
| 855 | 0,6672 | 0,6561 | 0,01117 | 0,02729 | ns | >0,9999 |
| 860 | 0,6736 | 0,6622 | 0,01145 | 0,02729 | ns | >0,9999 |
| 865 | 0,6805 | 0,6688 | 0,01169 | 0,02729 | ns | >0,9999 |
| 870 | 0,6874 | 0,6758 | 0,01163 | 0,02729 | ns | >0,9999 |
| 875 | 0,6943 | 0,6827 | 0,01163 | 0,02729 | ns | >0,9999 |
| 880 | 0,7010 | 0,6893 | 0,01174 | 0,02729 | ns | >0,9999 |
| 885 | 0,7073 | 0,6954 | 0,01187 | 0,02729 | ns | >0,9999 |
| 890 | 0,7128 | 0,7007 | 0,01218 | 0,02729 | ns | >0,9999 |
| 895 | 0,7174 | 0,7049 | 0,01247 | 0,02729 | ns | >0,9999 |
| 900 | 0,7213 | 0,7086 | 0,01266 | 0,02729 | ns | >0,9999 |
| 905 | 0,7243 | 0,7114 | 0,01297 | 0,02729 | ns | >0,9999 |
| 910 | 0,7263 | 0,7129 | 0,01339 | 0,02729 | ns | >0,9999 |
| 915 | 0,7277 | 0,7140 | 0,01363 | 0,02729 | ns | >0,9999 |
| 920 | 0,7300 | 0,7163 | 0,01374 | 0,02729 | ns | >0,9999 |
| 925 | 0,7349 | 0,7210 | 0,01385 | 0,02729 | ns | >0,9999 |
| 930 | 0,7426 | 0,7285 | 0,01405 | 0,02729 | ns | >0,9999 |
| 935 | 0,7526 | 0,7383 | 0,01426 | 0,02729 | ns | >0,9999 |
| 940 | 0,7641 | 0,7495 | 0,01459 | 0,02729 | ns | >0,9999 |
| 945 | 0,7766 | 0,7614 | 0,01515 | 0,02729 | ns | >0,9999 |
| 950 | 0,7901 | 0,7744 | 0,01567 | 0,02729 | ns | >0,9999 |
| 955 | 0,8056 | 0,7893 | 0,01634 | 0,02729 | ns | >0,9999 |
| 960 | 0,8235 | 0,8065 | 0,01696 | 0,02729 | ns | >0,9999 |
| 965 | 0,8402 | 0,8231 | 0,01711 | 0,02729 | ns | >0,9999 |
| 970 | 0,8514 | 0,8340 | 0,01744 | 0,02729 | ns | >0,9999 |
| 975 | 0,8556 | 0,8380 | 0,01759 | 0,02729 | ns | >0,9999 |
| 980 | 0,8542 | 0,8375 | 0,01673 | 0,02729 | ns | >0,9999 |
| 985 | 0,8494 | 0,8332 | 0,01614 | 0,02729 | ns | >0,9999 |
| 990 | 0,8413 | 0,8256 | 0,01571 | 0,02729 | ns | >0,9999 |
| 995 | 0,8338 | 0,8185 | 0,01535 | 0,02729 | ns | >0,9999 |
